# Irradiation Can Selectively Kill Tumor Cells while Preserving Erythrocyte Viability in a Co-Culture System

**DOI:** 10.1371/journal.pone.0127181

**Published:** 2015-05-27

**Authors:** Ming Gong, Jin-Ting Yang, Yun-Qing Liu, Li-Hui Tang, Yin Wang, Lie-Ju Wang, Feng-Jiang Zhang, Min Yan

**Affiliations:** 1 Department of Anesthesiology, the Second Affiliated Hospital, Zhejiang University School of Medicine, Hangzhou 310009, China; 2 Jiangsu Province Key Laboratory of Anesthesiology, Xuzhou Medical College, Xuzhou 221004, China; ENEA, ITALY

## Abstract

An understanding of how to safely apply intraoperative blood salvage (IBS) in cancer surgery has not yet been obtained. Here, we investigated the optimal dose of ^137^Cs gamma-ray irradiation for killing human hepatocarcinoma (HepG2), gastrocarcinoma (SGC7901), and colonic carcinoma (SW620) tumor cells while preserving co-cultured erythrocytes obtained from 14 healthy adult volunteers. HepG2, SGC7901, or SW620 cells were mixed into the aliquots of erythrocytes. After the mixed cells were treated with ^137^Cs gamma-ray irradiation (30, 50, and 100 Gy), tumor cells and erythrocytes were separated by density gradient centrifugation in Percoll with a density of 1.063 g/ml. The viability, clonogenicity, DNA synthesis, tumorigenicity, and apoptosis of the tumor cells were determined by MTT assay, plate colony formation, 5-ethynyl-2'-deoxyuridine (EdU) incorporation, subcutaneous xenograft implantation into immunocompromised mice, and annexin V/7-AAD staining, respectively. The ATP concentration, 2,3-DPG level, free Hb concentration, osmotic fragility, membrane phosphatidylserine externalization, blood gas variables, reactive oxygen species levels, and superoxide dismutase levels in erythrocytes were analyzed. We found that ^137^Cs gamma-ray irradiation at 50 Gy effectively inhibited the viability, proliferation, and tumorigenicity of HepG2, SGC7901, and SW620 cells without markedly damaging the oxygen-carrying ability or membrane integrity or increasing the oxidative stress of erythrocytes *in vitro*. These results demonstrated that 50 Gy irradiation in a standard ^137^Cs blood irradiator might be a safe and effective method of inactivating HepG2, SGC7901, and SW620 cells mixed with erythrocytes, which might help to safely allow IBS in cancer surgery.

## Introduction

Oncologic surgery is often accompanied by massive blood loss that requires blood transfusion [1]. Allogeneic blood transfusion (ABT) is often used to replenish blood lost during tumor surgery. However, ABT in oncological surgery is associated with many complications, such as postoperative infection, pulmonary complications, poor outcomes, and even the promotion of tumor recurrence [1]. To minimize or prevent the complications of ABT, intraoperative blood salvage (IBS) was recently developed. This technique involves the suction, collection, filtration, and washing of blood from the surgical field before reinfusion with erythrocytes. The technique has been widely used in a variety of surgeries and very effectively saves blood and reduces ABT-related complications [2].

However, IBS has long been viewed as contraindicated in cancer surgery for fear that free tumor cells in the shed blood could be concentrated, spread and metastasized during IBS [3]. Free tumor cells can be easily detected in the salvaged blood in diverse cancer surgeries such as gynecologic oncology [4], urologic oncology [5–7] and hepatocellular carcinoma operations [8, 9]. These free tumor cells mixed in the salvaged blood originate primarily from the surgical field, and they can proliferate, enrich a subpopulation, and even develop a new carcinoma [10]. Furthermore, it has been shown that patients infused with salvaged blood containing abundant tumor cells had worse outcomes and reduced survival, whereas those infused with salvaged blood containing no or low tumor cells had a better outcome and significantly longer tumor-free survival time [11]. Therefore, the risk of metastatic potential can only be prevented by eliminating the tumor cells from IBS before reinfusion.

The autotransfusion device alone insufficiently removes contaminating tumor cells from the salvaged blood. Leukocyte depletion filtration (LDF) and gamma irradiation are the two main methods for removing tumor cell contamination. Some studies have shown that LDF is a very effective technique for clearing tumor cells in the blood salvaged during gynecologic oncology surgery [4] or prostate cancer surgery [12] or for killing cancer cell lines that were seeded in blood [13, 14]. However, at a high tumor cell load (e.g., 2×10^7^ /200 ml), LDF cannot completely remove all the tumor cells [14], which is consistent with the previous statement that LDF is not safe to allow the re-transfusion of salvaged blood containing 10^7^ or more tumor cells during oncologic surgery [11]. This limitation of LDF was further confirmed by the fact that a single LDF step could not completely remove all tumor cells in cases with tumors that unexpectedly ruptured during surgery, even after two consecutive filtrations [8]. Patients with unexpected tumor rupture during surgery might have a load of tumor cells in the salvaged blood that is higher than the capacity of LDF (e.g., more than 2×10^7^ /200 ml), but the exact number of tumor cells remains unclear. Another crucial threat is the hypotension triggered by the LDF-treated blood [15, 16], which might be associated with the elevation of bradykinin [16] and interleukin-6 [17] during the transfusion of salvaged blood treated by LDF. In addition, LDF also markedly decreased the ATP content in erythrocytes [18]. Thus, Hansen’s group advocated gamma irradiation to eliminate tumor-laden blood and found that 50 Gy irradiation was sufficient to inactivate tumor cells in blood shed during cancer surgery with a reduction rate exceeding 10 logs [19]. In the last 6 years, more than 700 cancer patients at 30 different tumor centers in Europe were safely infused with the salvaged blood treated by irradiation [11]. In contrast, cytokeratin-19, a biomarker of tumor cells, was detected in the salvaged blood from the cancer patient using RT-PCR even though this salvaged blood was treated with 100 Gy irradiation [13]. Thus, the question whether gamma irradiation efficiently eliminates tumor cells from the salvaged blood is under debate.

The aims of this study were to estimate the efficiency of ^137^Cs gamma-ray irradiation in eliminating human hepatocarcinoma (HepG2), gastrocarcinoma (SGC7901), and colonic carcinoma (SW620) cell lines mixed with erythrocytes *in vitro* and *in vivo* and to determine the effect of irradiation on erythrocytes salvaged from Chinese persons.

## Materials and Methods

### Ethical statement

All human experimental protocols were approved by the Ethics Committee of 2^nd^ Affiliated Hospital, Zhejiang University School of Medicine, on May 29, 2013. Written informed consent was obtained from 14 healthy adult volunteers. The blood from healthy volunteers was mixed with 200 ml of heparin in a normal saline solution at 30 U/ml. The washed erythrocyte concentrates (250–300 ml) from a single donor were prepared using a Cell Saver 5 (Haemonetics Co, Boston, MA, USA). The hematocrit of concentrated erythrocytes was 40–60%.

All animal experimental protocols were strictly conducted in accordance with the Declaration of the National Institutes of Health Guide for Care and Use of Laboratory Animals (Publication No. 80–23, revised 1996) and monitored and approved by the Laboratory Animal Care Committee of Zhejiang University.

### Study design

Eight erythrocyte samples from 8 volunteers were used in subcutaneous xenograft experiments *in vivo*, and 6 erythrocyte samples from 6 volunteers were used in other experiments *in vitro*. After the washed erythrocytes were collected from healthy adult volunteers by the Cell Saver 5, each erythrocyte sample from 14 volunteers was divided into 3 aliquots that were mixed with HepG2, SGC7901, or SW620 cells. The final concentration of tumor cells in each aliquot was 5×10^5^ /ml. A single batch of each cell line was subdivided and mixed with the 14 different erythrocyte samples. Each portion of mixed cells was allocated into 4 groups: the control group (non-irradiated group) and 30, 50, and 100 Gy gamma-ray irradiation groups. The mixed cells were irradiated in a ^137^Cs blood irradiator (Gammacell3000 Elan, Atomic Energy of Canada Ltd., Chalk River, ON, Canada). Dosimetry showed that a central dose rate was 9.75 Gy/min. After irradiation, tumor cells were isolated by single-step density gradient centrifugation in Percoll (GE Healthcare Bio-Sciences AB, Uppsala, Sweden). The density of Percoll was 1.063 g/ml, which provides a significantly greater separation of cancer cells from the blood with maximal reduction in leukocyte contamination [10]. Then, the viability of separated tumor cells was determined by MTT assay, plate colony formation, 5-ethynyl-2'-deoxyuridine (EdU) incorporation, subcutaneous xenografting into immunocompromised mice, and annexin V/7-AAD staining. Meanwhile, ATP, 2,3-DPG, free Hb concentration, osmotic fragility, membrane phospholipid bilayer integrity, blood gas variables, reactive oxygen species (ROS), and superoxide dismutase (SOD) in separated erythrocytes were assayed. For xenograft tumor experiments, 32 male immunocompromised BALB/c nude mice were randomly divided into 4 groups xenotransplanted with 0, 30, 50, and 100 Gy irradiated HepG2 cells (*n* = 8). Another 64 mice were xenotransplanted with SGC7901 and SW620 cells in the same groupings (*n* = 8). The total number of immunocompromised mice was 96.

### Animals

Ninety-six SPF male BALB/c nude mice (18–20 g, six weeks old) were purchased from Shanghai Laboratory Animal Center, Chinese Academy of Sciences (Shanghai, China). Mice were housed in sterile and static micro-isolation cages to be fed on irradiated standard pellet chow and sterile water *ad libitum* in a 12-hour light/dark cycle and room temperature of 22±1°C. All cages contained sterile wood shavings, bedding and a cardboard tube for environmental enrichment. All mice were acclimatized for one week before the experiment.

### Cell lines

Human hepatocarcinoma (HepG2), gastrocarcinoma (SGC7901), and colonic carcinoma (SW620) cell lines were purchased from the cell bank of the Shanghai Branch of the Chinese Academy of Sciences. HepG2 cells were cultured in high glucose DMEM supplemented with 10% fetal bovine serum (FBS). SGC7901 cells were cultured in RPMI-1640 medium with 15% FBS. SW620 cells were cultured in Leibovitz's L15 medium with 20% FBS. Media for cell culture were supplemented with 100 U/ml penicillin and 100 mg/ml streptomycin, at 37°C in a humidified incubator containing 5% CO_2_. All media and FBS were purchased from Gibco (Grand Island, NY, USA).

### MTT assay

Each group of isolated tumor cells was seeded onto 96-well plates (10^3^ /well for 24 h and 48 h; or 5×10^2^ /well for 72 h) in quintuplicate at 37°C in a humidified incubator containing 5% CO_2_. The cell viability was evaluated by the amount of viable cells stained by 3-(4,5-dimethylthiazol-2-yl)-2,5-diphenyltetrazolium bromide (MTT, Sigma-Aldrich Inc, St Louis, MO, USA), which was released with dimethylsulfoxide (DMSO, Sigma-Aldrich Inc, St Louis, MO, USA). The optical density was detected at 490 nm with an automatic microplate reader (BioRad, Hercules, CA, USA). The killing effect of irradiation on tumor cell lines was calculated as cell viability, which was indicated as follows: (the percentage of viable cells) = (absorbance of treated wells—absorbance of blank wells) / (absorbance of the control wells—absorbance of blank wells) × 100%.

### Colony formation assay

Each group of isolated tumor cells was seeded onto 6-well plates in triplicate at a density of 2×10^2^ /well for the control groups and 10^4^ /well for the irradiated groups, and the medium was exchanged every 2 days. After incubating for 14 days, cells were fixed with methanol and stained with Giemsa. The colony formation rate was calculated by dividing the number of colonies by the number of seeded cells [20].

### EdU incorporation assay

Each group of isolated tumor cells was seeded onto 96-well plates in triplicate at a density of 10^3^ /well for 24 h of incubation or 5×10^2^ /well for 72 h of incubation. The cells were incubated for an additional 2 h in respective medium containing 50 μM EdU (RiboBio, Guangzhou, China). Cells were then washed with PBS, fixed and permeabilized with PBS containing 4% paraformaldehyde and 0.5% triton X-100. Cells were incubated with 1× Apollo reaction cocktail (100 μl/well) for 30 min. DNA was incubated with Hoechst 33342 stain (100 μl/well) for 30 min and visualized with an inverted fluorescence microscope (Leica DM5500, Germany). For each EdU experiment, five random fields were imaged by 100× magnification. Captured images were processed and analyzed with ImageJ software. The number of EdU positive cells was identified by Hoechst nuclei staining and expressed as a percentage of the total number of cells in each field [21].

### Tumor cell apoptosis assay

Each group of isolated tumor cells was washed once with cold PBS and once in annexin V binding buffer and then stained with annexin V and 7-AAD (BD Biosciences Pharmingen, San Diego, CA, USA) for 15 min in the dark at 25°C. Cell apoptosis was detected and analyzed by BD FACSCalibur Flow Cytometry.

### Xenograft tumor models

The immunocompromised mice were anesthetized with 50 mg/kg sodium pentobarbital and xenotransplanted with 2×10^6^ cells (HepG2, SGC7901 or SW620) in 200 μl in the left flank near the upper extremity [20]. The tumor volume (*V*) was calculated weekly by measurement (caliper rule) of tumor length and width according to the formula *V* = π/6×a×b^2^ (a>b). After 8 weeks, mice were euthanized by CO_2_, and xenograft tumors were excised for measuring final dimensions and weights. The tumor tissues were then fixed for hematoxylin and eosin (H&E) staining.

### Assay of ATP, 2,3-DPG and free Hb concentrations

Immediately after irradiation, the free Hb concentration in the solution of erythrocytes and the ATP and 2,3-diphosphoglycerate (2,3-DPG) concentrations in the erythrocytes were measured using Quantitative Human Competitive ELISA kits (Hermes Criterion Biotechnolog, Canada) according to the manufacturer’s instructions.

### Osmotic fragility test

The osmotic fragility of erythrocytes was analyzed according to a previous study [22]. Immediately after irradiation, separated erythrocytes were incubated in a series of solutions with NaCl ranging from 0.24% to 0.68% as the final concentration. The absorbance at 540 nm was measured with an ultraviolet spectrophotometer to indicate hemoglobin concentration in the supernatant. The erythrocytes treated with normal saline were used as a negative control (0% hemolysis), and those treated with distilled water were used as a positive control (100% hemolysis).

### Assay of phospholipid bilayer integrity

Immediately after irradiation, the physical integrity of the membrane phospholipid bilayer in erythrocytes was evaluated by the amount of phosphatidylserine on the outside of the cell membrane. Erythrocytes were washed three times with cold PBS and once in annexin V binding buffer and then stained with PE–annexin V (BD Biosciences Pharmingen, San Diego, CA, USA) for 15 min in the dark at 25°C. The phosphatidylserine externalization was assayed by BD FACSCalibur Flow Cytometry [23].

### Assay of blood gas variables

Immediately after irradiation, the extracellular K^+^ and Na^+^ concentration, pH, Hb, PO_2_ (partial pressure of oxygen dissolved in the solution), and P_50_ (the PO_2_ at which hemoglobin is half-saturated with oxygen) values in the erythrocyte solution were measured using an automated blood gas analyzer (Roche Diagnostics Cobas b123, Mannheim, Germany).

### Assay of ROS

Before irradiation, erythrocytes were incubated with 10 μM CM-H_2_-DCFDA (Invitrogen, Carlsbad, CA) for 30 min at 37°C with 50 μM H_2_O_2_ (positive control) or without H_2_O_2_ [24]. Immediately after irradiation, erythrocytes were washed once with warm PBS, and the fluorescence intensity was detected by BD FACSCalibur Flow Cytometry at 488 nm excitation/530 nm emission.

### Assay of SOD

Immediately after irradiation, SOD in erythrocytes was measured by a commercial kit (Nanjing Jiancheng Bioengineering Institute, Nanjing, China) following the manufacturer’s instructions as previously described [25].

### Statistical analysis

Data are shown as the means ± SEM. The data for colony formation, tumor volume, and osmotic fragility were analyzed using the unpaired Student’s *t* test. All other data were analyzed by one-way ANOVA following the Newman-Keuls test. All statistical analyses were performed using GraphPad Prism Version 5.0 (GraphPad Prism Software, San Diego, CA, USA). A value of *P*<0.05 was considered statistically significant.

## Results

### ^137^Cs gamma-ray irradiation inhibited the viability of tumor cell lines *in vitro*

**Fig 1:**
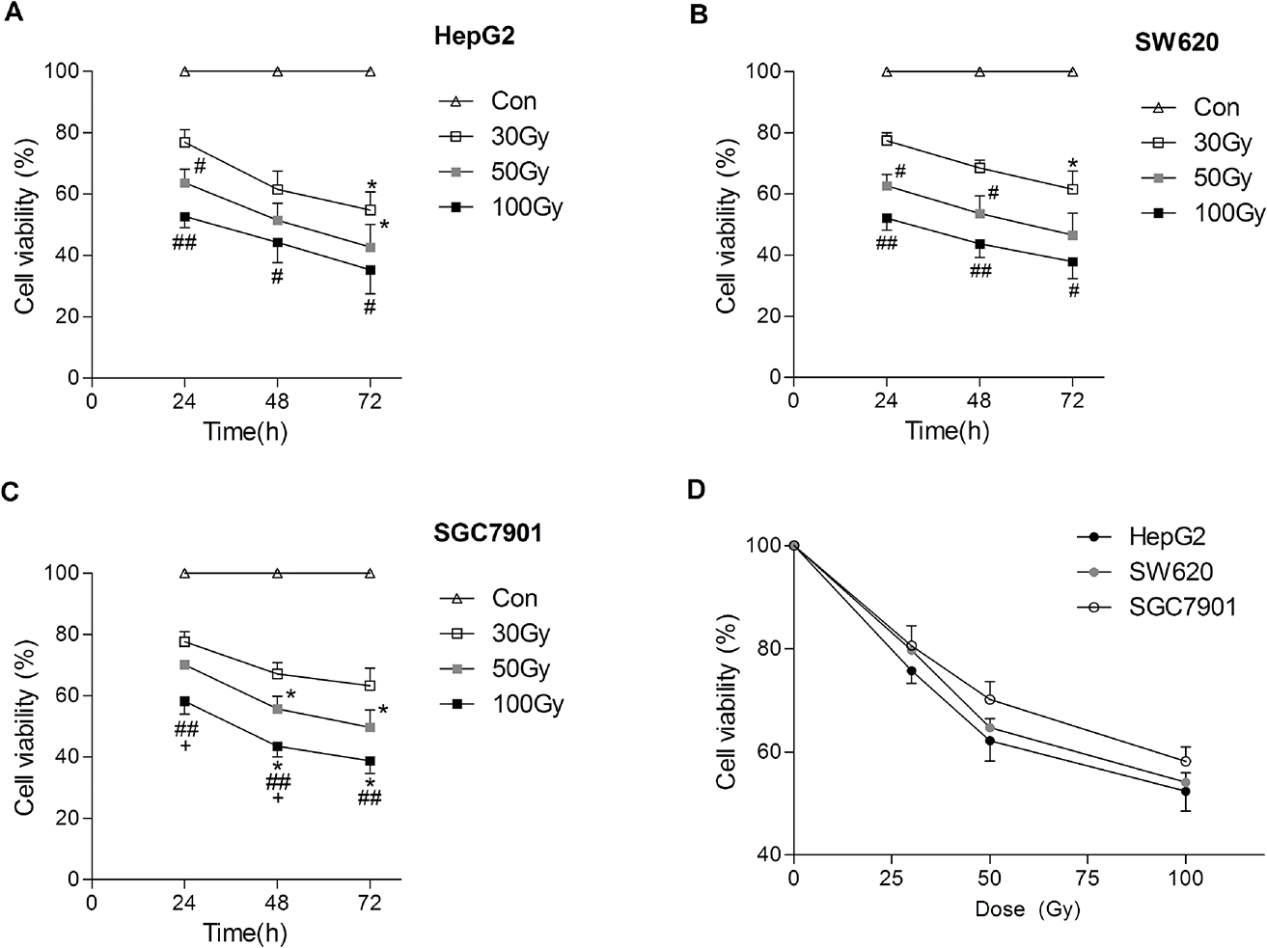
^137^Cs gamma-ray irradiation inhibits the viability of tumor cell lines *in vitro*. After ^**137**^Cs gamma-ray irradiation (0, 30, 50 and 100 Gy), HepG2 (A), SW620 (B), and SGC7901 (C) cells separated from human erythrocytes were cultured for 24 h, (D) 48 h and 72 h, and then the viability was detected by MTT assay. The cell viability was indicated by the percentage of viable cells. Date are means ± SEM; *n* = 6 erythrocyte samples from 6 volunteers in each group. Con: the dose of irradiation was 0. *P<0.05 vs. the same treated group cultured for 24 h; ^**#**^ *P*<0.05, ^**##**^ *P*<0.01 vs. 30 Gy irradiation group at the same culture time; ^**+**^ *P*<0.05 vs. 50 Gy irradiation group at the same culture time.

**Fig 2:**
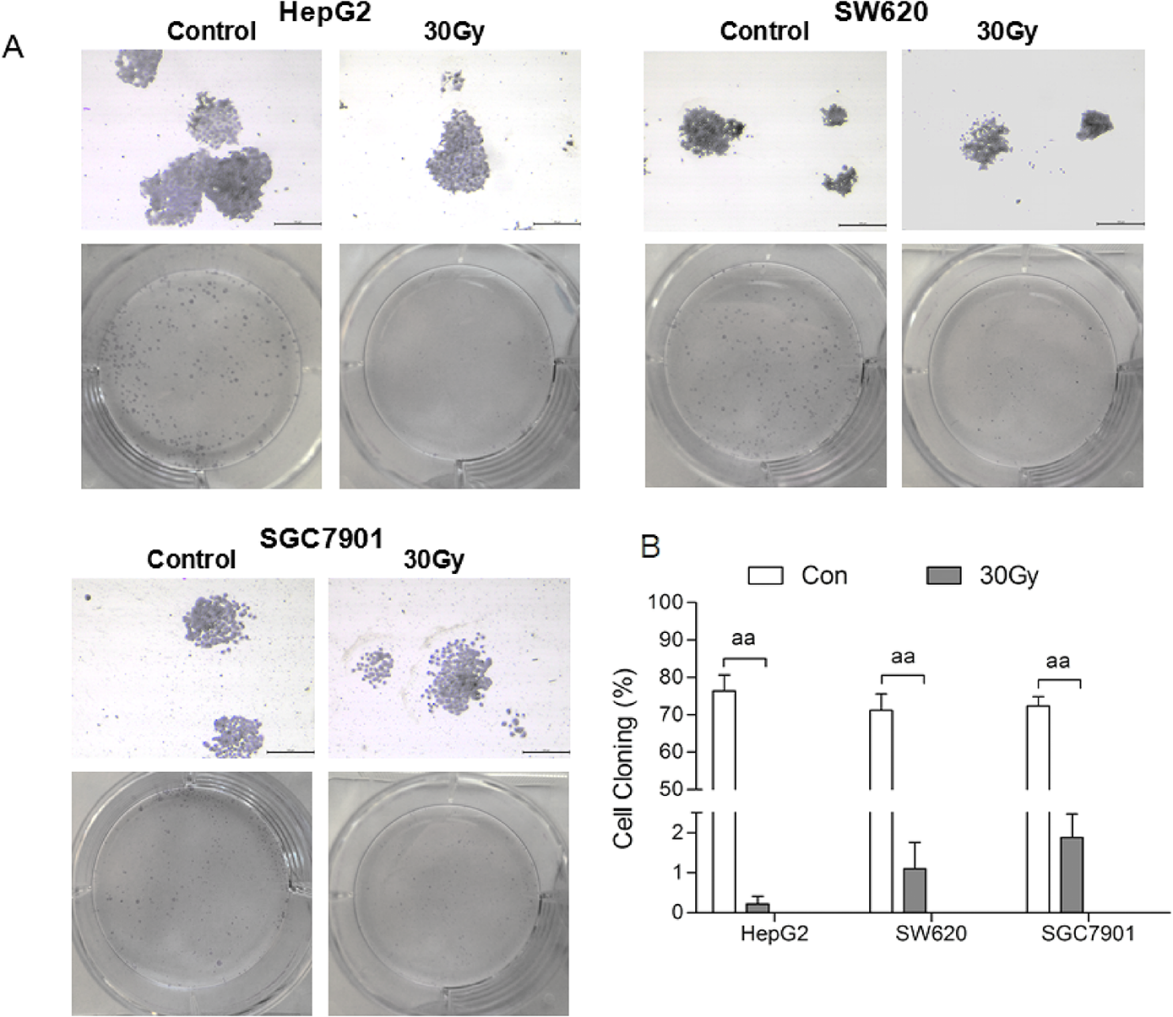
^137^Cs gamma-ray irradiation inhibits the colony formation by tumor cell lines *in vitro*. After ^**137**^Cs gamma-ray irradiation (0, 30, 50 and 100 Gy), HepG2, SW620 and SGC7901 cells separated from human erythrocytes were cultured for 14 d, and then the colony formation was detected by Giemsa staining. Representative Giemsa staining (scale bar: 100 μm) of HepG2, SW620 and SGC7901 cells 7 d after irradiation (A). Colony formation rate of tumor cells at 14 d after irradiation (B). Data are means ± SEM; *n* = 6 erythrocyte samples from 6 volunteers in each group. ^**aa**^ *P*<0.01 vs. Con (the dose of irradiation was 0 Gy).

**Fig 3:**
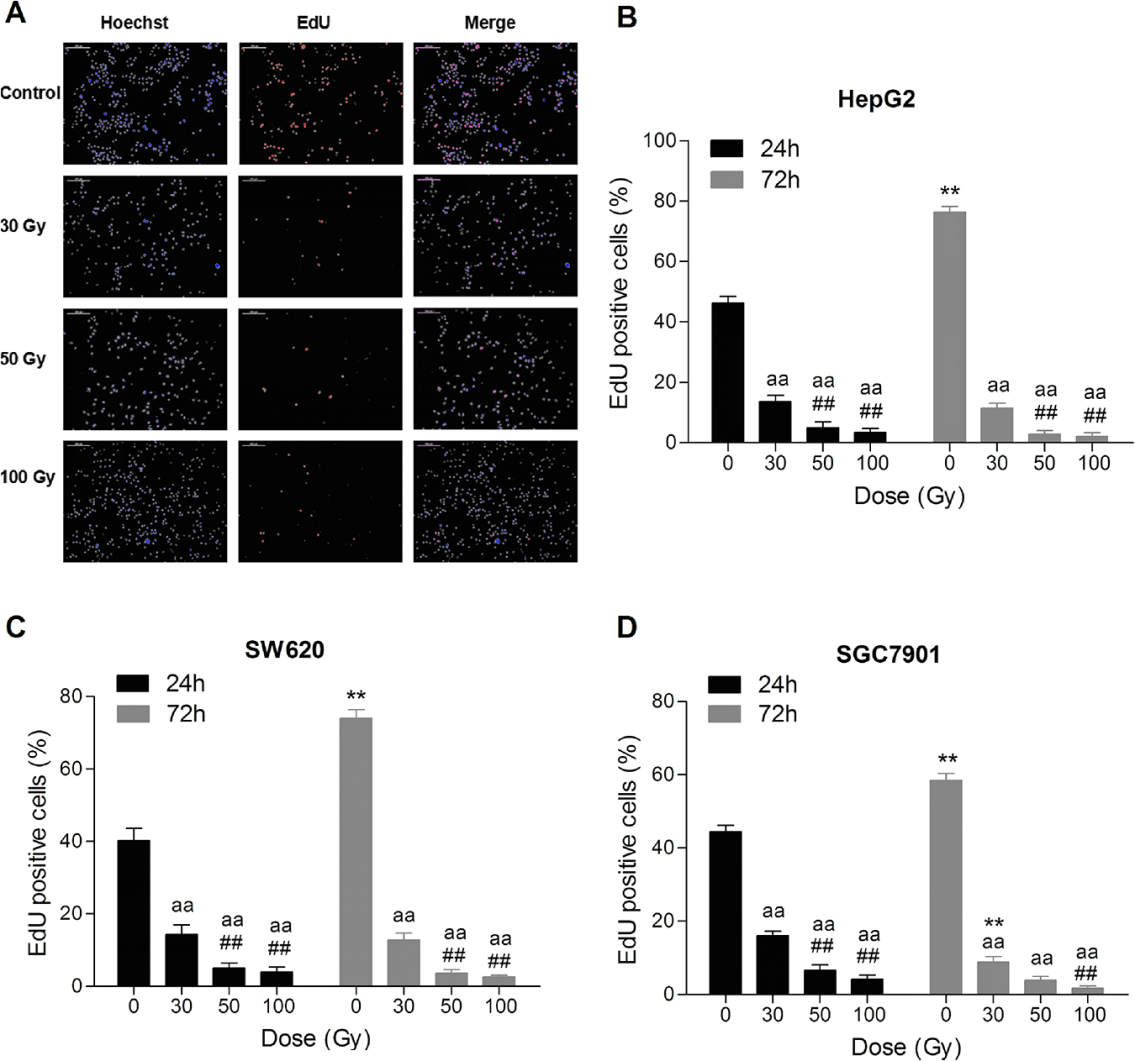
^137^Cs gamma-ray irradiation inhibits DNA synthesis in tumor cell lines *in vitro*. After ^**137**^Cs gamma-ray irradiation (0, 30, 50 and 100 Gy), HepG2, SW620 and SGC7901 cells separated from human erythrocytes were cultured for 24 h, and then DNA synthesis was detected by 5-ethynyl-2'-deoxyuridine (EdU) incorporation. Representative DNA synthesis (scale bar: 200 μm) of HepG2 cells at 24 h after irradiation with 0, 30, 50 and 100 Gy (A). DNA synthesis in HepG2 (B), SW620 (C) and SGC7901 (D) cells at 24 h and 72 h after irradiation. Date are means ± SEM; *n* = 6 erythrocyte samples from 6 volunteers in each group. **P<0.01 vs. the same treated group culturing for 24 h; ^**##**^ *P*<0.01 vs. 30 Gy irradiation group at the same culture time; ^**aa**^ *P*<0.01 vs. Con (the dose of irradiation was 0 Gy) at the same incubation time after treatment.

The viability, colony formation and DNA synthesis of HepG2, SGC7901 and SW620 cells subjected to ^137^Cs gamma-ray irradiation were significantly inhibited in a time- and dose-dependent manner (Figs 1–3). MTT assays indicated that HepG2 cells were more sensitive to gamma-ray irradiation compared with SW620 and SGC7901 cells (Fig 1); these results were in agreement with the colony formation analysis and EdU incorporation assays (Figs 2 and 3). Cell viability and DNA synthesis were significantly inhibited in all irradiated groups in a time-dependent manner (Figs 1 and 3). As shown in Fig 3, the DNA synthesis detected by the EdU incorporation assay in all three cell lines subjected to 100 Gy irradiation was still observed even after culturing for 72 h. Consistent with cell viability and DNA synthesis, irradiation significantly reduced colony formation in all three tumor cell lines. Colony formation in all three tumor cell lines was markedly reduced by 30 Gy irradiation (Fig 2). The minimal rate of reduction by 30 Gy irradiation, the ratio of the number of treated tumor cells in the assay to the number of untreated cells resulting in colony formation [19], ranged from 1.6 to 2.5 logs (S1 Table). After 50 and 100 Gy irradiation, no colony formation was found for HepG2 cells, and the minimal rate of reduction was more than 3.9 log (S1 Table). However, slight colony formation was still found for SGC7901 and SW620 cells (0.03±0.03%, 0.01±0.01%, respectively) subjected to 50 Gy irradiation after 14 days. Although SGC7901 cells were treated with 100 Gy irradiation, colony formation (0.03±0.03%) was still observed after 14 d.

### ^137^Cs gamma-ray irradiation inhibited the growth of xenograft tumors in immunocompromised mice

**Fig 4:**
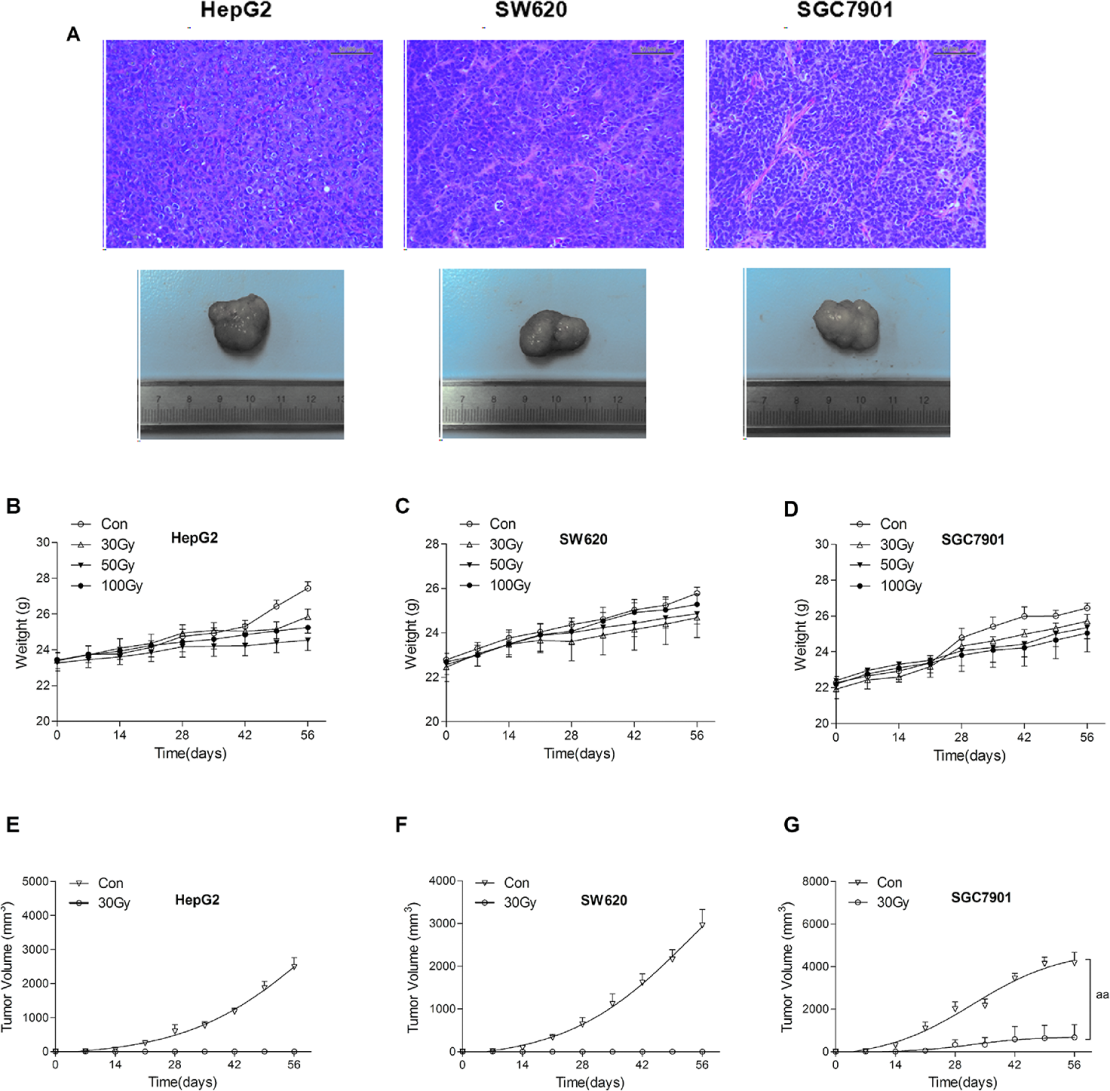
^137^Cs gamma-ray irradiation inhibits the growth of xenograft tumors in immunocompromised mice. After ^**137**^Cs gamma-ray irradiation (0, 30, 50 and 100 Gy), HepG2, SW620 and SGC7901 cells separated from human erythrocytes were subcutaneously xenotransplanted into male BALB/c nude mice. The histopathology of xenograft tumors growing up to 2 cm in diameter was detected by H&E staining. Representative subcutaneous xenograft tumor and histopathology of xenograft tumor developed by non-irradiated HepG2, SW620 and SGC7901 cells (scale bar: 50 μm) with H&E staining (A). The body weights of immunocompromised mice subcutaneously xenotransplanted with HepG2 (B), SW620 (C) and SGC7901 (D) cells. The volume of xenograft tumors developed by HepG2 (E), SW620 (F) and SGC7901 (G) cells subjected to 0 and 30 Gy irradiation in immunocompromised mice. Date are means ± SEM; *n* = 8 mice in each group. ^**aa**^ *P*<0.01 vs. Con (the dose of irradiation was 0 Gy).

All non-irradiated HepG2, SW620, and SGC7901 cells subcutaneously xenotransplanted into immunocompromised mice developed xenograft tumors (8/8 mice), which was confirmed by histopathology (Fig 4A). The volume of xenograft tumors that developed from non-irradiated tumor cells time-dependently increased in immunocompromised mice (Fig 4E–4G). HepG2 and SW620 cells subjected to 30 Gy irradiation did not develop xenograft tumors in any of 8 immunocompromised mice (Fig 4E and 4F). However, xenograft tumors developed from SGC7901 cells treated with 30 Gy irradiation, but the volume of these tumors was significantly decreased compared with the control group (Fig 4G). None of the three cell lines treated with 50 Gy irradiation developed xenograft tumors (0/8 mice in each group), and this result was the same for the 100 Gy irradiation treated groups (data not shown). The body weights of immunocompromised mice increased in a time-dependent manner after xenotransplantation with tumor cell lines, and there was no significant difference of body weights between the control group and irradiated group (Fig 4B–4D).

### ^137^Cs gamma-ray irradiation induced death in tumor cells *in vitro*

**Fig 5:**
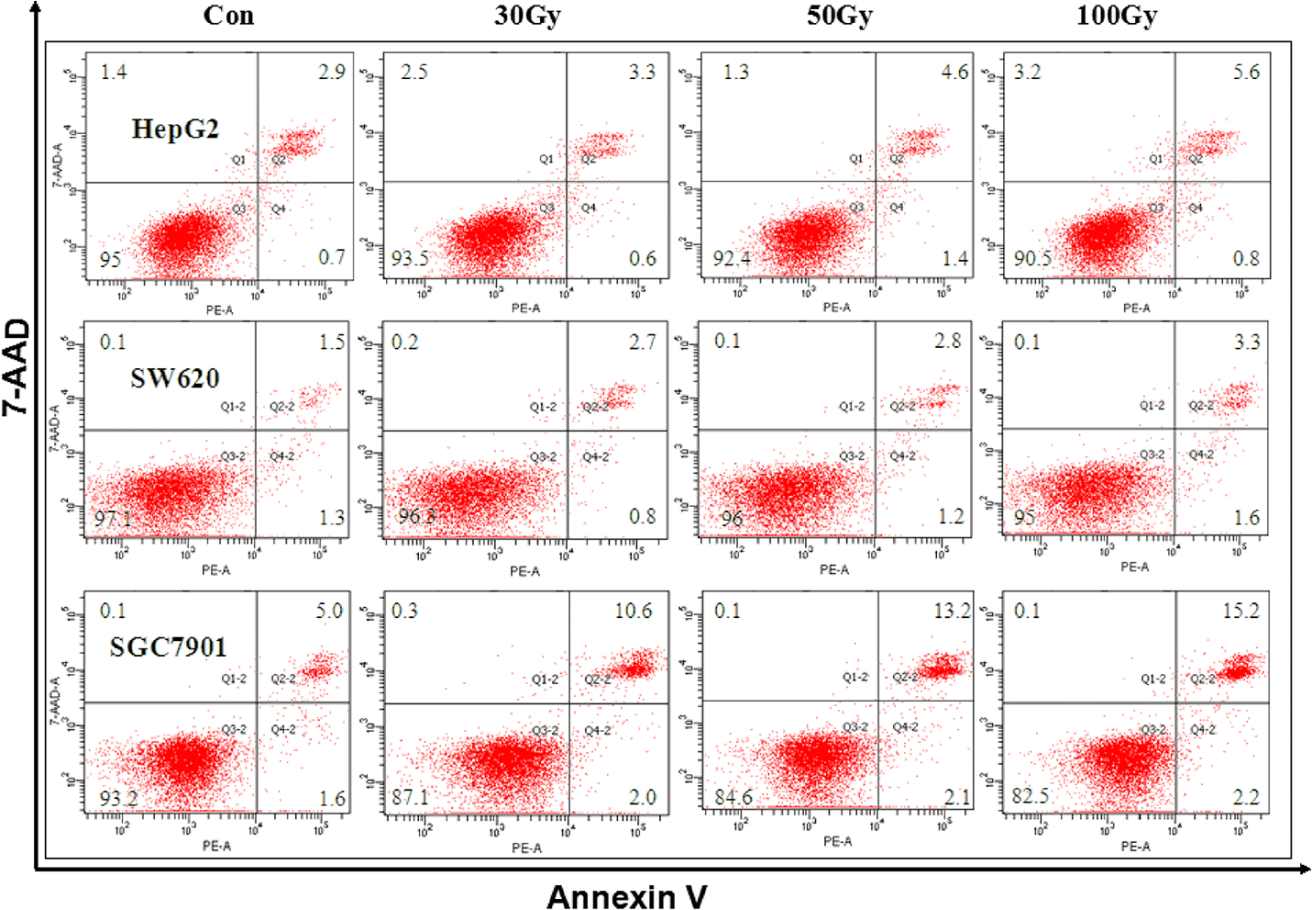
^137^Cs gamma-ray irradiation induces apoptosis and necrosis in tumor cell lines *in vitro*. Immediately after ^**137**^Cs gamma-ray irradiation (0, 30, 50 and 100 Gy), apoptosis and necrosis in HepG2, SW620 and SGC7901 cells separated from human erythrocytes was determined by annexin V/7-AAD staining.

**Fig 6:**
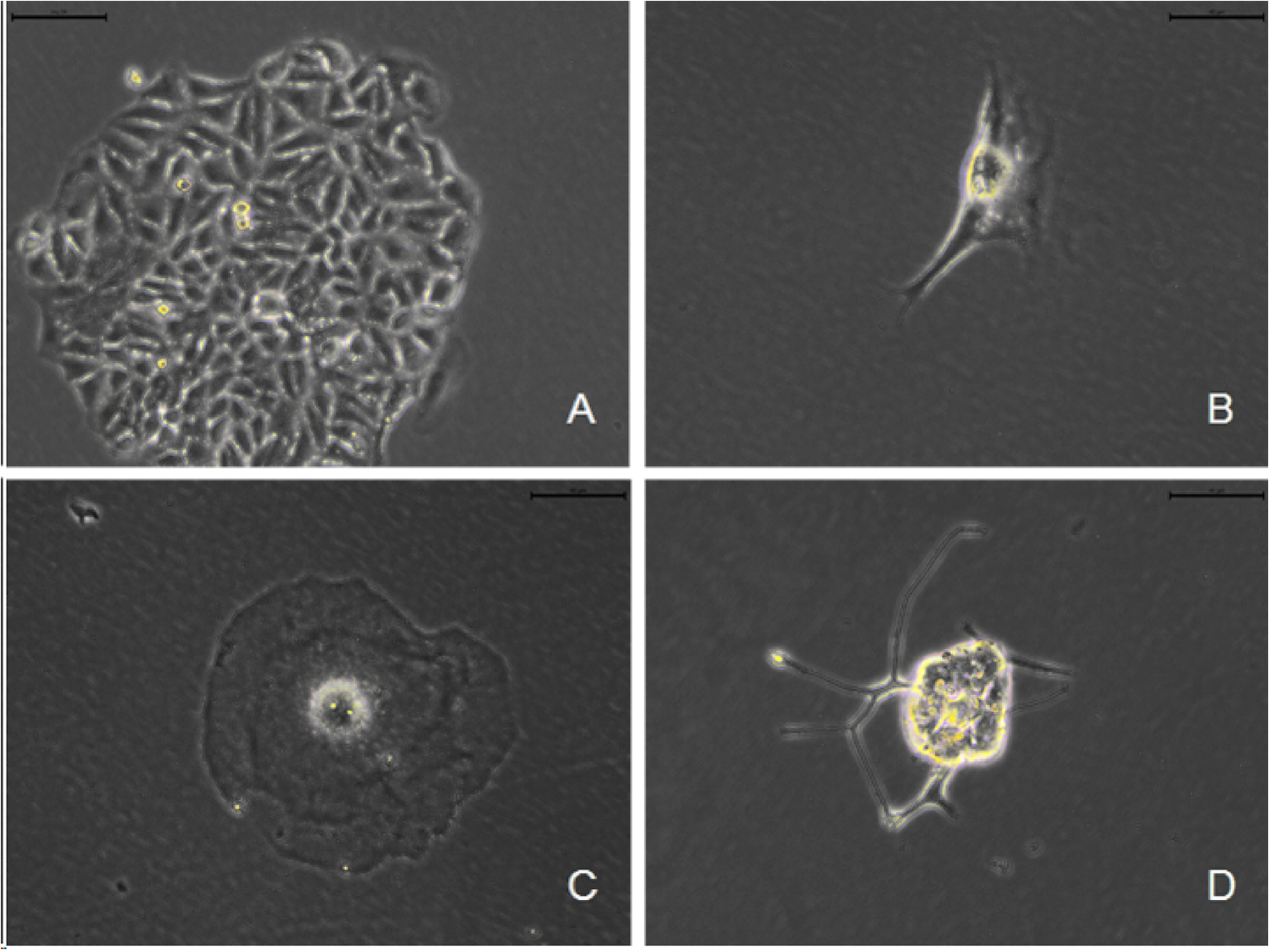
^137^Cs gamma-ray irradiation induces death in HepG2 cells *in vitro*. After 0 (A), 30 (B), 50 (C) and 100 (D) Gy ^**137**^Cs gamma-ray irradiation, HepG2 cells separated from human erythrocytes were cultured for 7 d, and then their morphology (scale bar: 40 μm) was observed.

Flow cytometry showed that cell death increased in a dose-dependent manner in response to ^137^Cs gamma-ray irradiation, and necrosis was the predominant form of cell death (Fig 5). This result was consistent with the morphology of HepG2 cells that were irradiated and then allowed to grow for 7 days; these cells showed cellular swelling, rupture of the cell membrane, and leakage of cytoplasmic contents (Fig 6, data from SW620 cells and SGC7901 cells not shown).

### Effects of ^137^Cs gamma-ray irradiation on erythrocytes *in vitro*

**Fig 7:**
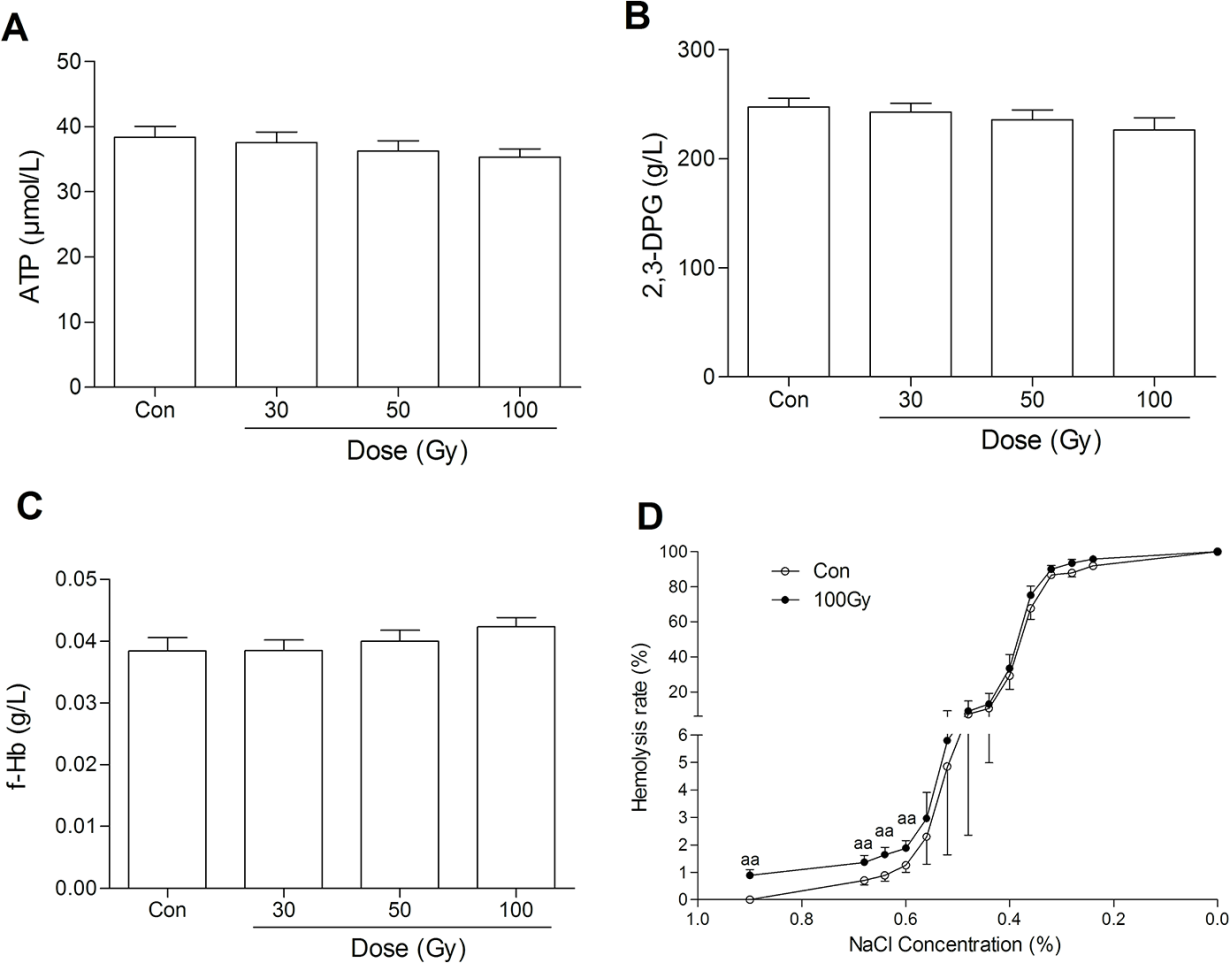
Effects of ^137^Cs gamma-ray irradiation on erythrocytes *in vitro*. After ^**137**^Cs gamma-ray irradiation (0, 30, 50 and 100 Gy), ATP (A), 2,3-DPG (B), free Hb (C) and osmotic fragility (D) in erythrocytes were determined. Data are means ± SEM; *n* = 14 erythrocyte samples from 14 volunteers in each group. ^**aa**^ *P*<0.01 vs. Con (the dose of irradiation was 0 Gy).

**Fig 8:**
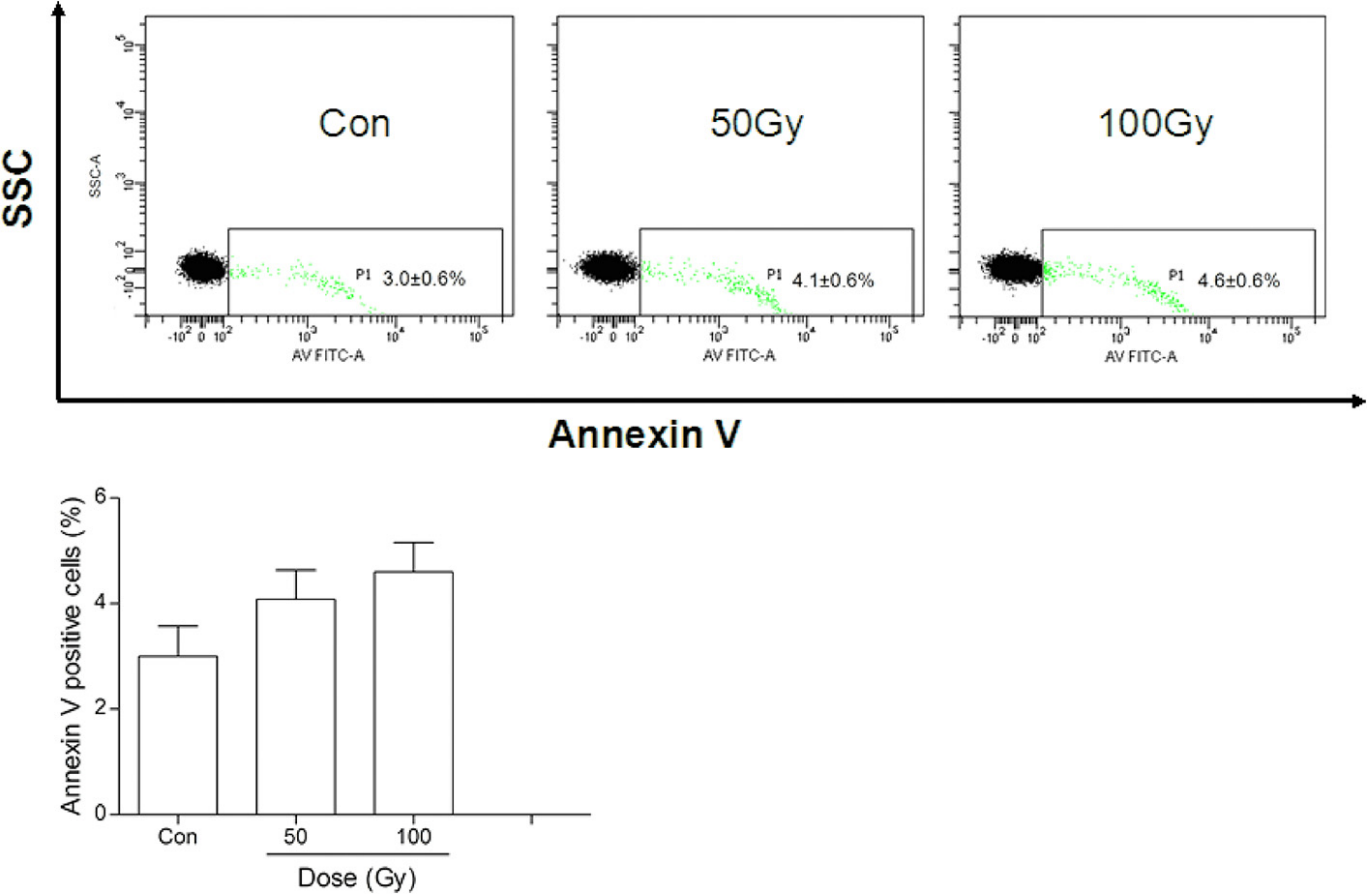
Effects of ^137^Cs gamma-ray irradiation on phosphatidylserine exposure in erythrocytic membranes. After ^**137**^Cs gamma-ray irradiation (0, 50 and 100 Gy), phosphatidylserine exposure in erythrocytic membranes was detected by annexin V staining. Data are means ± SEM; *n* = 14 erythrocyte samples from 14 volunteers in each group.

ATP, 2,3-DPG (a biomarker of oxygen-carrying function), and free Hb in erythrocytes were not significantly reduced by ^137^Cs gamma-ray irradiation (*P*>0.05, Fig 7A–7C). Osmotic fragility and extracellular K^+^ and Na^+^ in erythrocytes were all significantly altered by 100 Gy irradiation but not by 30 Gy irradiation compared with the control group (*P*<0.01, *P*<0.05 and *P*<0.01, Fig 7D and Table 1). The amount of phosphatidylserine on the outside of erythrocytes was not altered by irradiation (*P*>0.05, Fig 8). Similarly, other blood gas variables, such as pH, PO_2_, P_50_, and Cl^-^ in erythrocytes were all unaffected by irradiation (*P*>0.05, Table 1). In addition, extracellular K^+^ was markedly increased by 50 Gy irradiation compared with the control group (*P*<0.05, Table 1), which was not beyond the normal clinical range.

**Table 1:** Effects of ^137^Cs gamma-ray irradiation on blood gas variables in erythrocytes.

	Con	30 Gy	50 Gy	100 Gy
Hb (g/L)	129.84±10.21	129.59±10.72	126.93±10.39	130.26±10.42
pH	7.56±0.036	7.55±0.037	7.55±0.036	7.47±0.089
P_50_ (mmHg)	19.78±1.18	19.61±1.71	24.31±3.23	24.23±2.59
PO_2_ (mmHg)	187.72±5.34	190.96±5.54	190.28±6.14	188.92±5.72
K^+^(mmol/L)	3.06±0.37	4.22±0.49	4.80±0.60^a^	5.59±0.60^aa^
Na^+^ (mmol/L)	145.76±0.97	144.51±1.02	143.54±0.99	142.54±0.81^a^
Cl^—^(mmol/L)	139.06±1.55	139.38±1.49	139.46±1.52	139.55±1.50

After ^137^Cs gamma-ray irradiation (0, 30, 50 and 100 Gy), blood gas variables in erythrocytes was detected. Data are means ± SEM, *n* = 14 erythrocyte samples from 14 volunteers in each group.

^a^ *P*<0.05,

^aa^ *P*<0.01 vs. Con (the dose of irradiation was 0 Gy)

### Effects of ^137^Cs gamma-ray irradiation on ROS and SOD in erythrocytes *in vitro*

**Fig 9:**
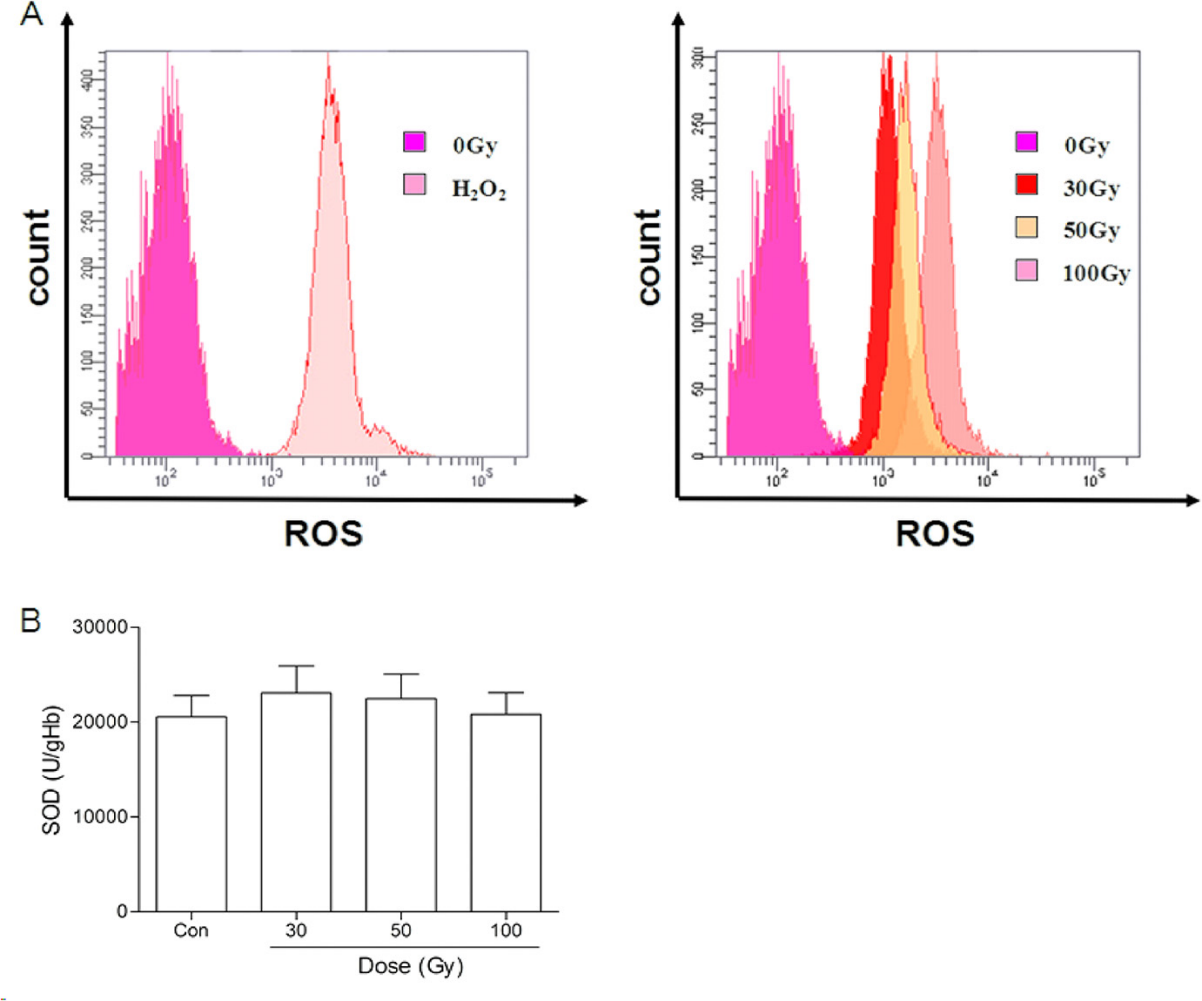
Effects of ^137^Cs gamma-ray irradiation on ROS levels and SOD in erythrocytes. After ^**137**^Cs gamma-ray irradiation (0, 30, 50 and 100 Gy), the ROS level (A) and SOD (B) in erythrocytes were determined by the fluorescence probe CM-H_2_-DCFDA and a commercial kit, respectively. Con: the dose of irradiation was 0 Gy; H_2_O_2_: positive control group. *n* = 14 (B) SOD of RBCs change after irradiation. Data are means ± SEM, *n* = 14.

As shown in Fig 9A, the ROS level indicated by the fluorescence probe CM-H_2_-DCFDA was sharply elevated in the positive control group (H_2_O_2_) compared with the control group (non-irradiated group), and ROS generation was elevated in a dose-dependent manner after ^137^Cs gamma-ray irradiation. However, SOD was not significantly altered in any of the irradiated groups compared with the control group (Fig 9B).

## Discussion

Stomach cancer, liver cancer and colon cancer are three of the most common gastrointestinal cancers and the leading causes of gastrointestinal cancer death throughout the world [26]. Especially in China, the incidence of these three tumors is rapidly increasing [26]. Gastrointestinal cancer has been effectively treated by surgery. However, gastrointestinal cancer surgery is associated with a high likelihood of allogeneic blood transfusion, which produces many complications [27]. In addition, allogeneic blood shortages are widespread [28] and are particularly critical in China [29]. All these adverse events hinder the promotion of gastrointestinal cancer surgery. How to safely use IBS instead of allogeneic blood transfusion during cancer surgery is becoming a hot topic in the clinic.

In this study, we found that treating mixed cells (5×10^5^ /ml cell lines in erythrocytes) with ^137^Cs gamma-ray irradiation dose-dependently inhibited the cell viability, DNA synthesis, and colony forming ability of HepG2, SGC7901 and SW620 cells. The colony formation of HepG2 cells was completely inhibited by 50 Gy and 100 Gy irradiation, and the colony formation of SW620 cells was completely inhibited by 100 Gy irradiation. The xenograft model in immunocompromised mice further confirmed that the tumorigenicity of all three tumor cell lines was completely inhibited by 50 Gy and 100 Gy irradiation but not by 30 Gy irradiation. Additionally, 30 and 50 Gy irradiation did not significantly influence the membrane integrity, osmotic fragility, or oxygen-carrying function of erythrocytes. However, the osmotic fragility and extracellular K^+^ in erythrocytes were significantly increased by 100 Gy irradiation. We suggest that 50 Gy irradiation may be a safe and effective method to maximally inactivate (especially in inhibiting tumorigenicity) gastrointestinal cancer cells such as HepG2, SGC7901, and SW620 during IBS.

Cell colony formation and DNA synthesis measured by EdU incorporation are commonly used to evaluate the proliferative capacity of malignant cells. The xenotransplantation of cancer cells into immunocompromised mice provides the ability to assess cell tumorigenicity [30]. Our results showed that 50 and 100 Gy irradiation markedly inhibited the proliferative capacity of HepG2, SGC7901, and SW620 cells. Although slight colony formation was still observed in SGC7901 and SW620 cells subjected to 50 or 100 Gy irradiation, HepG2 colony formation was completely inhibited by 50 or 100 Gy irradiation after culturing for 14 d. This finding was consistent with a previous study showing that no cell colony formation was observed after the irradiation of tumor cell-contaminated blood with 50 Gy [19]. None of these three tumor cell lines subjected to 50 Gy and 100 Gy irradiation developed xenograft tumors in immunocompromised mice, indicating that the tumorigenicity of HepG2, SGC7901, and SW620 cells separated from erythrocytes was very mild after 50 Gy and 100 Gy irradiation.

We found that irradiation dose-dependently increased cell death in the three tumor cell lines. However, necrosis, but not apoptosis, played a key role in the response to irradiation (30, 50, 100 Gy) in tumor cells, which is confirmed by their morphology. Our data were supported by previous reports showing that high doses of radiation (32–50 Gy) directly induced necrosis in neurons [31] and in dividing cells such as tumor cells [32]. Necrosis in these three tumor cell lines caused by ^137^Cs gamma-ray irradiation may be attributed to mitotic catastrophe due to DNA destruction, chromosomal aberrations and dysfunction of cell cycle checkpoints [33].

Eukaryotic DNA is the most important molecular target of gamma-ray irradiation, and thus it should have limited effects on enucleated erythrocytes, in theory. One important biomarker of the oxygen-carrying function of erythrocytes is 2,3-DPG, which is also susceptible to IBS procedure [34]. In addition, P_50_ is an index of oxygen affinity and reflects the oxygen-carrying capacity [35]. Oxygen affinity is influenced by temperature, pH, ATP and 2,3-DPG in erythrocytes [35]. Therefore, preserving the ATP and 2,3-DPG concentrations and the P_50_ in erythrocytes might be vital to the application of IBS in cancer surgery. Our study showed that ATP, 2,3-DPG, free Hb, pH, P_50_ and PO_2_ in erythrocytes were not significantly affected by 30, 50, or 100 Gy irradiation, which is consistent with previous studies [36, 37]. The release of K^+^ was dose-dependently increased by irradiation. Although the extra-erythrocytic K^+^ concentration was increased to 4.80 ± 0.59 mmol/L by 50 Gy irradiation, it was still within the normal clinical range (3.5–5.5 mmol/L). However, osmotic fragility significantly increased along with the marked elevation of extra-erythrocytic K^+^ and the reduction of extra-erythrocytic Na^+^ by 100 Gy irradiation, suggesting that excess irradiation might induce serious hemolysis of erythrocytes [22].

In addition, we found that the phosphatidylserine externalization in irradiated erythrocytes was not significantly increased, even at irradiation intensities up to 100 Gy. Phosphatidylserine externalization in erythrocytes reflects the physical disintegration of the membrane phospholipid bilayer, which commonly occurs in senescent and dead cells and is attributed to both metabolic depletion and oxidative modification [23, 38]. Irradiation-induced ROS generation impairs the erythrocyte membrane integrity and accelerates cell aging [39] [40]. We measured the ROS levels in erythrocytes subjected to different doses of ^137^Cs gamma-ray irradiation by the fluorescence probe CM-H_2_-DCFDA and found that the intracellular ROS levels increased upon irradiation in a dose-dependent manner. The ROS levels in erythrocytes induced by 100 Gy irradiation reached the same level that was induced by H_2_O_2_ (positive control). However, erythrocytic SOD activities in all groups were not significantly different, suggesting that the preserved antioxidative capacity might be beneficial to protect erythrocytes against oxidative stress injury, such as membrane phosphatidylserine externalization, induced by irradiation.

In general, it is suggested that the irradiation of murine blood samples spiked with tumor cells and the subsequent injection of these samples into mice might most effectively demonstrate whether the irradiation procedures proposed can suppress metastasis and preserve erythrocyte function. This transfusion model was not performed, and this may be a limitation of the present work. However, the biological alterations associated with murine erythrocyte dysfunction are different from those of human erythrocytes [41–43]. Therefore, this suggested experiment is not necessarily the most pertinent. In the current work, we used subcutaneous xenografts in immunocompromised mice to confirm whether our irradiation procedures could effectively inactivate tumor cells *in vivo* after the proliferation capacity of tumor cells was determined *in vitro* by conventional methods, such as the MTT, colony formation and DNA synthesis assays [19–21]. The xenotransplantation of tumor cells into immunocompromised mice is a well-known method to effectively determine cell tumorigenicity [30]. We acknowledge that the effect of blood irradiation on erythrocytes should be evaluated not only *in vitro* but also *in vivo*. The transfusion of these mixed erythrocytes subjected to blood irradiation into the immunocompromised mice might better approximate clinical settings to estimate the effect of irradiation on erythrocytes. Although it is well accepted that not all strains of immunocompromised mice are appropriate in this transfusion model and screening a feasible strain of immunocompromised mice is a difficult task [44, 45], our next key project should be the irradiation of human (or murine) blood samples spiked with tumor cells and then injecting these into mice on the basis of this study.

In conclusion, our present study suggested that 50 Gy irradiation given by a standard ^137^Cs blood irradiator is a safe and effective method to inactivate HepG2, SGC7901, and SW620 cells mixed with erythrocytes while preserving the quality of erythrocytes. The application of 50 Gy irradiation might help to safely introduce IBS into cancer surgery, but more *in vivo* preclinical studies to evaluate the safety and validity are required.

## Supporting Information

S1 ARRIVE Checklist The animal research: reporting in vivo experiments. (PDF)

S1 Table Clonogenic survival in tumor cell lines subjected to blood irradiation. After blood irradiation by ^137^Cs gamma-ray (0, 30, 50 and 100 Gy), HepG2, SW620 and SGC7901 cells separated from erythrocytes were cultured for 14 d, and then the colony formation was detected by Giemsa staining. a: Number of colonies formed/number of cells cultured; b: Number of colonies formed/number of tumor cells added to blood before irradiation.
(DOC)
