# Supplementary material for: Irradiation Can Selectively Kill Tumor Cells while Preserving Erythrocyte Viability in a Co-Culture System
Source: PLoS One. 2015 May 27;10(5):e0127181. doi: 10.1371/journal.pone.0127181 (PMC4446348; doi:10.1371/journal.pone.0127181)
Supplement: S1 Table — After blood irradiation by 137Cs gamma-ray (0, 30, 50 and 100 Gy), HepG2, SW620 and SGC7901 cells separated from erythrocytes were cultured for 14 d, and then the colony formation was detected by Giemsa staining. a: Number of colonies formed/number of cells cultured; b: Number of colonies formed/number of tumor cells added to blood before irradiation. (DOC) [file pone.0127181.s002.doc]

**S_1_ Table Clonogenic survival in tumor cell lines subjected to blood irradiation**

|  | Colony formation | | | | Demonstrated log reduction | | |
| --- | --- | --- | --- | --- | --- | --- | --- |
|  | Non-irradiated blood^a^ | Irradiated blood^b^ | | |  |  |  |
|  |  | 30Gy | 50 Gy | 100 Gy | 30 Gy | 50 Gy | 100 Gy |
| HepG2 | 152.8 ±4.2  /200 | 22.2/  10^4^ | 0/  10^4^ | 0/  10^4^ | 2.5 | >3.9 | >3.9 |
| SW620 | 142.5 ± 4.3  /200 | 188/  10^4^ | 1/  10^4^ | 0/  10^4^ | 1.6 | 3.9 | >3.9 |
| SGC7901 | 144.7 ± 2.5  /200 | 188.3/  10^4^ | 3/  10^4^ | 3/  10^4^ | 1.6 | 3.4 | 3.4 |

After blood irradiation by ^137^Cs gamma-ray (0, 30, 50 and 100 Gy), HepG2, SW620 and SGC7901 cells separated from erythrocytes were cultured for 14 d, and then the colony formation was detected by Giemsa staining.

a: Number of colonies formed/number of cells cultured; b: Number of colonies formed/number of tumor cells added to blood before irradiation.
